# Preoperative neutrophil-to-lymphocyte ratio behaves as an independent prognostic factor even in patients with postoperative complications after curative resection for gastric cancer

**DOI:** 10.1007/s00423-022-02432-9

**Published:** 2022-01-08

**Authors:** Jaume Tur-Martínez, Javier Osorio, Noelia Pérez-Romero, Noelia Puértolas-Rico, Manuel Pera, Salvadora Delgado, Joaquín Rodríguez-Santiago

**Affiliations:** 1 Service of General Surgery, University Hospital Mútua Terrassa, Terrassa, Barcelona, Spain; 2 Department of Surgery, Universitat Autònoma de Barcelona, Bellaterra, Spain; 3 Section of Gastrointestinal Surgery. Hospital del Mar, Universitat Autónoma de Barcelona, Hospital del Mar Medical Research Institute (IMIM), Barcelona, Bellaterra Spain

**Keywords:** Gastric cancer, Neutrophil-to-lymphocyte ratio, Preoperative systemic inflammation, Postoperative complications

## Abstract

**Purpose:**

The aim of this study was to determine if the prognostic value of the preoperative neutrophil-to-lymphocyte ratio (NLR) could be modified by the presence of postoperative complications (POC) and their severity in patients with gastric adenocarcinoma resected with curative intent.

**Methods:**

A retrospective study based on a prospective database of patients with resectable gastric adenocarcinoma treated with radical intention (R0) between January 1998 and February 2012. The primary endpoint was overall survival according to preoperative peripheral blood NLR and postoperative complications. Clinicopathological variables, preoperative blood tests, POC and its severity (Clavien–Dindo classification), type of POC (infectious or not infectious) and mortality were registered. A univariate and multivariate analysis (step forward Cox regression) was performed. The Kaplan–Meier method was used to assess overall survival.

**Results:**

The 147 patients with gastric cancer who had undergone radical resection were included from an initial cohort of 209 patients. Univariant analysis: type of surgery, pT, pN, postoperative complications (Clavien–Dindo ≥ 3) and preoperative NLR ≥ 2.4 were significantly associated with survival (*p* < 0.05). Patients with POC showed worse long-term survival (*p* = 0.000), with no difference (*p* = 0.867) between infectious or non-infectious POC. NLR ≥ 2.4 was associated with infectious POC (*p* < 0.001). Patients with preoperative NLR ≥ 2.4 (*p* = 0.02) had a worse prognosis. Multivariate analysis: pN (*p* < 0.001), postoperative complications (*p* < 0.001) (HR 3.04; 95% CI: 1.97–4.70) and NLR ≥ 2.4 (*p* = 0.04) (HR = 1.55; 95% CI: 1.02–2.3) were independent prognostic factors.

**Conclusion:**

The preoperative inflammatory state of patients with gastric cancer measured by NLR behaves as an independent prognostic factor, even in patients with POC.

## Introduction

It is known that preoperative systemic inflammatory response is related to the development, progression and invasion of tumour cells and consequently to overall survival [1] in most tumours.

Different biological parameters have been identified as markers of a systemic inflammatory state. Some of the most relevant markers are neutrophil-to-lymphocyte ratio (NLR) [2], lymphocyte-to-monocyte ratio (LMR) [3], platelets-to-lymphocytes ratio (PLR) [4], Nutritional Prognosis Index ([10 × albumin] + [0,005 × lymphocytes]) [5], Glasgow Prognosis Score (a combination of C reactive protein and albumin values) [6], systemic immune-inflammatory index (neutrophils × platelets/lymphocytes) [7] and derived neutrophil-to-lymphocyte ratio (neutrophil to white blood cells – neutrophils) [8]. The most frequently analysed biomarker to evaluate systemic inflammatory response is NLR, as it is a simple, easy-to-use and inexpensive tool.

Several studies have shown that a high preoperative neutrophil-to-lymphocyte ratio (NLR) value in peripheral blood tests represents an independent prognostic factor of overall survival in different types of tumours, including gastric cancer [3, 9].

It has also been proven that postoperative complications (especially severe and infectious ones) are independent prognostic factors for long-term survival after curative gastrectomy [10–12].

Likewise, preoperative systemic inflammatory response and postoperative complications are both going to affect overall survival, but at different moments of the disease. The preoperative systemic inflammatory state is influenced by the tumour immunity and may induce anti- or pro-tumour response. The presence of postoperative complications (especially more infectious ones) could activate different pro-tumour pathways and impair survival [13].

Given the influence on the survival of preoperative systemic inflammation and systemic inflammation following postoperative complications, both factors should be analysed together to ensure the independent predictive value of each parameter.

The aim of this study was to determine if the prognostic value of the preoperative NLR could be influenced by the presence of postoperative complications in patients with gastric adenocarcinoma resected with curative intent.

## Methods

### Data collection

This is a retrospective observational study using a prospectively maintained database including all patients treated in the University Hospital Mútua Terrassa (Barcelona, Spain) with gastric adenocarcinoma undergoing gastric resection with curative intent (R0) from January 1998 to February 2012. The study was approved by the ethical committee of our hospital (*Acta 10/2019*). Exclusion criteria are age under 18 years old, chronic inflammatory or autoimmune illness, neoadjuvant chemotherapy and/or radiotherapy, R1 and R2 resections, intraoperative finding of carcinomatosis and palliative surgery. The preoperative study included gastro-oesophageal endoscopy, thorax-abdomen-pelvis CT and blood tests almost two weeks before surgery. The following preoperative data were systematically collected: age, gender, preoperative ASA grade classification (American Society Anaesthesiologist), neutrophils, lymphocytes, neutrophil-to-lymphocyte ratio (the optimal cut-off value for NLR was obtained according to the median value of the cohort in order to compare higher vs. lower index), tumour location (superior, middle, distal third or gastric linitis), type of gastrectomy (total, subtotal, or other) and type of lymphadenectomy (according to the Japanese Gastric Cancer Guidelines 2017) [14]. All cases were discussed in a multidisciplinary tumour board.

Resected specimens were analysed following the 7th Edition TNM classification [15]. Lauren classification (intestinal, diffuse or mixed) [16] was also specified. Patients with serosa invasion or positive lymph nodes (≥ pT_3_ and/or ≥ pN_1_) received adjuvant chemotherapy with mitomycin plus tegafur, according to our hospital protocol.

All postoperative complications (POC) occurring during the first 30 days after surgery were registered, and their severity was graded with the Clavien–Dindo (C-D) classification (grade I–II: minor complications; grade III–IV: major complications which required any interventional treatment or presented organ failure with intensive care support; and grade V: mortality) [17]. Complications were also divided into infectious and non-infectious. Infectious complications were classified according to the previous description published by our group [18]: Sepsis was defined as the presence of two or more systemic inflammatory response syndrome criteria. Intraabdominal infection was defined as (i) an abscess or diffused infection within the abdominal cavity diagnosed by ultrasound or computed tomography, with positive culture; (ii) the presence of an anastomotic leakage (defined as full-thickness gastrointestinal defect involving the anastomosis); (iii) duodenal stump fistula (bile or purulent drainage from a drain placed close to the duodenal stump); (iv) pancreatic fistula (drain output with amylase content) or (v) acute cholecystitis. Respiratory septic complications included pneumonia (defined as new or progressive infiltrate on chest X-ray, accompanied by fever, leukocytosis or leukopenia and purulent sputum, for which antibiotic treatment was started) and pleural empyema.

Wound infection was defined as a deep surgical site infection that required treatment with antibiotics agents or superficial wound drainage. Urinary tract infection was defined according to positive urine culture and clinical findings (fever, urgency, frequency, dysuria or suprapubic tenderness). Finally, catheter-related bloodstream infection was defined as bacteraemia/fungemia in a patient with an intravascular catheter with at least one positive blood culture obtained from a peripheral vein and no other apparent source for the bloodstream infection except the catheter.

Postoperative follow-up was done according to international guidelines [19, 20] with clinical examination 1 month after discharge, a general blood test with tumour markers and clinical examination every six months and annual abdominal CT scan and gastroscopy, until a 5-year follow-up was achieved for initial stages (pTis, pT1N0M0). All other stages were followed with gastroscopy and abdominal CT every six months during the first two years and then annually.

### Primary endpoint

The primary endpoint was overall survival, according to preoperative peripheral blood NLR and postoperative complications.

### Statistical analysis

Data are shown as frequencies and percentages for categorical variables or as median (interquartile range) for quantitative variables. Chi-square was used to analyse if the patients with higher preoperative NLR had suffered more postoperative complications and more severe complications. Overall survival was calculated from the date of surgery until death from any cause or the date of the last follow-up in living patients. A descriptive statistical analysis and a univariate and multivariable Step Forward Cox regression was performed for the prognostic factors. Kaplan–Meier curves according to NLR with the presence or not of POC were plotted and compared using log-rank statistics. The SPSS software package (SPSS Inc, Chicago, IL, USA), version 25, was used to manage patient data and perform statistical analyses.

## Results

### Cohort data

**Fig. 1:**
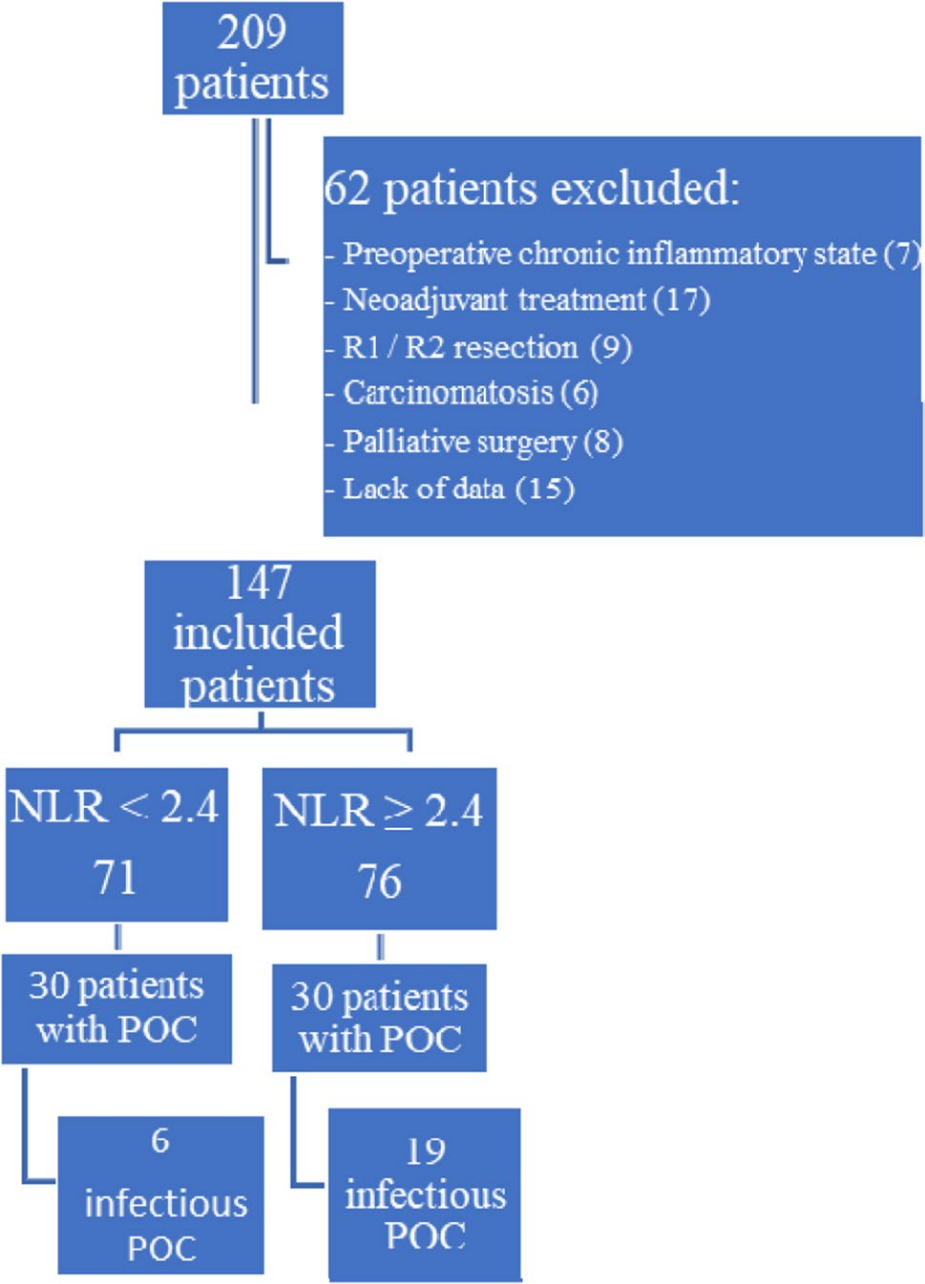
Flow-chart of patients’ selection. *NLR*, neutrophil-to-lymphocyte ratio; *POC*, postoperative complications

Of the 209 patients with resectable gastric cancers, 62 were excluded because they did not meet the inclusion criteria (47 patients) or because they lacked preoperative leukocyte count data (15 patients) (Fig. 1). A total of 147 patients were included for analysis, with a median follow-up of 43.9 months [1–204]. Table 1 shows the clinical and pathological variables of included patients: median age was 67.5 years [33–92], with a majority of men (65%). The most common ASA classifications were ASA-II (68%) and ASA-III (20%); 46% of tumours were distal third; most of the patients were treated with a subtotal gastrectomy and D2 lymphadenectomy (83%). Intestinal adenocarcinoma was the most prevalent Lauren histology type (48.3%), and most of the tumours were locally advanced. POC were registered in 60 patients (41%), 25 (17%) of them were infectious and 32 (22%) were severe (C-D ≥ 3). All POC are listed in Table 2, being most frequent: intraabdominal abscess (8.8%), anastomosis leak (8.8%) and pneumonia (5.4%). Thirty-six patients (24.5%) presented one POC, 18 (12.2%) patients had 2 and 6 (4%) patients had ≥ 3 POC.

**Table 1:** Descriptive analysis and patients’ distribution according to NLR values

Characteristics	*n*	%	NLR < 2.4	NLR ≥ 2.4	*p*
	147		*n* = 71 (%)	*n* = 76 (%)	
Gender					0.51
Male	96	65	48 (67.6)	48 (63.1)	
Women	51	35	23 (32.4)	28 (36.9)	
ASA grade					0.35
1	15	10.8	6 (8.4)	9 (11.8)	
2	94	68.1	50 (70.4)	44 (57.9)	
3	28	20.3	11 (15.5)	17 (22.4)	
4	1	0.7	1 (1.4)	0 (0)	
NC	9		3 (4.3)	6 (7.9)	
Tumour location					0.78
Distal 1/3	70	46	33 (46.5)	37 (48.7)	
Medial 1/3	48	32.7	24 (33.8)	24 (31.6)	
Upper 1/3	28	19	14 (19.7)	14 (18.4)	
Gastric linitis	1	0.7	0 (0)	1 (1.3)	
Type of surgery					0.33
Subtotal gastrectomy	100	68	48 (67.6)	52 (68.4)	
Total gastrectomy	42	28.6	19 (26.8)	23 (30.3)	
Others	5	3.4	4 (5.6)	1 (1.3)	
Lymphadenectomy					0.36
D2	122	83	61 (85.9)	61 (80.3)	
D1	25	17	10 (14.1)	15 (19.7)	
Lauren type					0.39
Intestinal	71	48.3	32 (45.1)	41 (53.9)	
Diffuse	44	29.9	26 (36.6)	18 (23.7)	
Mixed	30	20.4	13 (18.3)	17 (22.4)	
pT					0.48
pT1	16	10.9	8 (11.3)	8 (10.5)	
pT2	42	28.6	24 (33.8)	18 (23.7)	
pT3	78	53.1	33 (46.5)	45 (59.2)	
pT4	11	7.4	6 (8.4)	5 (6.6)	
pN					0.83
pN0	40	27.2	21 (29.6)	19 (25)	
pN1	30	20.4	14 (19.7)	16 (21)	
pN2	29	19.7	15 (21.1)	14 (18.5)	
pN3a	22	15	11 (15.5)	11 (14.5)	
pN3b	26	17.7	10 (14.1)	16 (21)	
Postoperative complications					0.69
No	87	59.2	41 (57.8)	46 (60.5)	
Yes	60	40.8	30 (42.2)	30 (39.5)	
C-D					0.63
0	87	59.2	40 (56.4)	47 (61.8)	
I–II	28	19.1	13 (18.3)	15 (19.7)	
III–IV	23	15.6	13 (18.3)	10 (13.2)	
V	9	6.1	5 (7)	4 (5.3)	
Infectious complications					< 0.001
No	35	23.8	25 (35.2)	10 (13.1)	
Yes	25	17	6 (8.4)	19 (25)	
Adjuvant Chemotherapy					
No	64	43.4	31 (43.7)	33 (43.4)	
Yes	83	56.6	40 (56.3)	43 (56.6)	

*NLR*, neutrophil-to-lymphocyte ratio; *C-D*, Clavien–Dindo classification; *ASA*, American Society of Anaesthesiologist; *NC*, not corresponded

**Table 2:** Description of non-infectious and infectious postoperative complications

Non-infectious (%)	Infectious (%)
Bleeding (3.4)	Intraabdominal abscess (8.8)
Paralytic ileus (3.4)	Anastomosis leak (8.8)
Respiratory failure (2.7)	Pneumonia (5.4)
Upper gastrointestinal bleeding (2.7)	Duodenal stump leak (3.4)
Anaemia (2)	Abdominal wall abscess (2)
Pancreatic fistula (2)	Wound infection (2)
Acute distress respiratory syndrome (2)	Urine infection (1.4)
Pleural effusion (1.4)	Others (4.1)
Delayed gastric emptying (1.4)	
Others (5.4)	

Patients with high preoperative NLR (*p* = 0.69) did not present more POC. The postoperative mortality rate at 30 days was 6.1%.

### Preoperative NLR results

Median preoperative leukocyte counts and ratios were neutrophils 4.2 × 10^9^/L (1.6–14.2), lymphocytes 1.8 × 10^9^/L (0.4–3.9) and NLR 2.4 (0.8–16.4). The 50th percentile of NLR (2.4) was used as a cut-off value. The sample was divided into two groups according to NLR value. Both groups had similar clinical characteristics, histological types and tumour stage, but infectious POC were more prevalent in the NLR ≥ 2.4 group (*p* < 0.001) (Table 1).

**Fig. 2:**
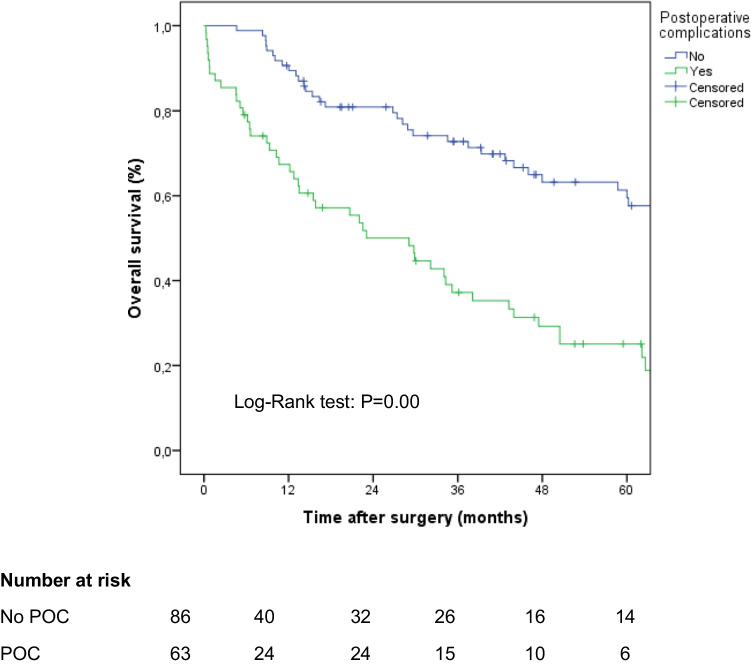
Kaplan–Meier curves comparing overall survival in patients with and without postoperative complications

Patients with POC showed worse long-term survival (*p* = 0.000) (Fig. 2), with no difference (*p* = 0.867) between infectious (29.1 months [4.10–54.13], 5-year overall survival 21.3 months) or non-infectious POCs (29.9 months [17.18–42.62], 5-year overall survival 25.6 months).

**Fig. 3:**
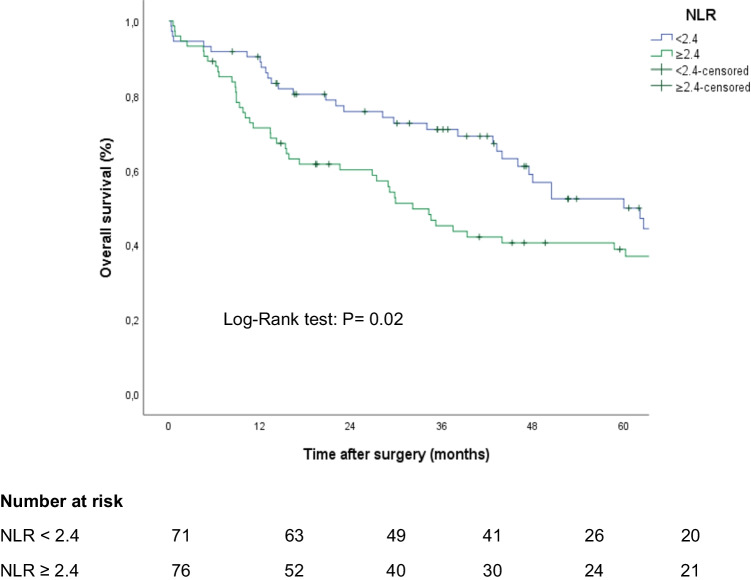
Kaplan–Meier curves comparing overall survival according to the neutrophil-to-lymphocyte ratio (NLR)

**Fig. 4:**
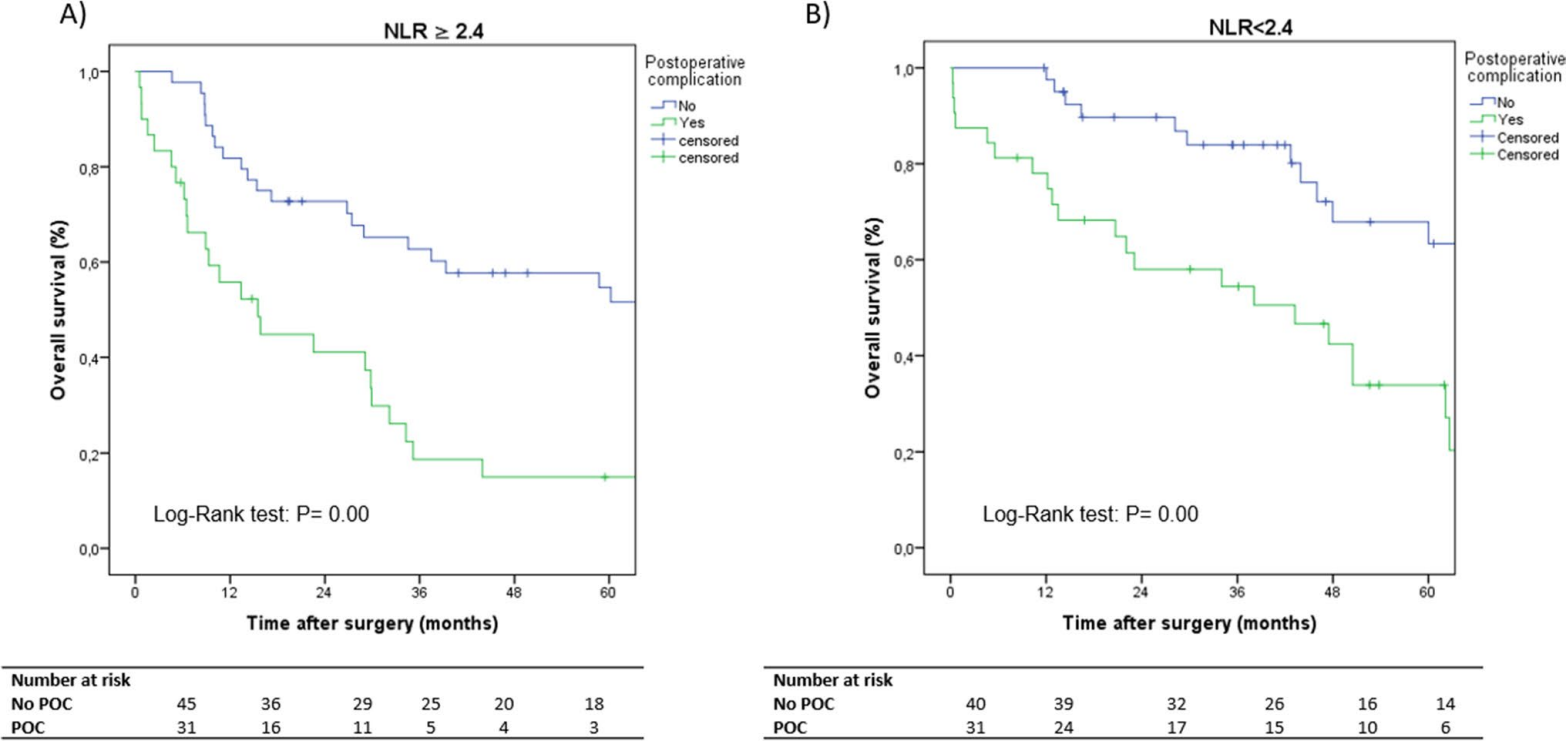
Kaplan–Meier curves comparing overall survival according to (**A**) NLR ≥ 2.4 with and without POC and (**B**) NLR < 2.4 with and without POC. *NLR*, neutrophil-to-lymphocyte ratio; *POC*, postoperative complications

The group of patients with preoperative NLR ≥ 2.4 (*p* = 0.02) also had a worse prognosis (Fig. 3). NLR maintained its prognostic relevance (*p* = 0.00) in patients with NLR < and ≥ 2.4, with and without POC (Fig. 4A, B). Similarly, NLR was also related to survival in the group of patients with stage III tumours (34.04 months [95% CI 19.05–49.02] vs. 15.37 months [95% CI 12.44–18.31], *p* = 0.01).

In the univariant survival analysis, factors related to worse long-term survival were the type of surgery, D2 lymphadenectomy, serosa invasion (pT3-4), nodal metastasis, the existence of POC (especially those classified as Clavien–Dindo ≥ 3) and preoperative NLR ≥ 2.4 (Table 3).

**Table 3:** Univariate and multivariate analyses of clinicopathological variables in relation to overall survival

Variables	Univariant		Multivariant	
	HR	*p*	HR	*p*
Age	1 (0.99–1.02)	0.82		
Gender		0.07		
Male	1			
Women	0.65 (0.40–1.04)			
Tumour location		0.07		
Distal 1/3	1			
Medial 1/3	1.23 (0.77–1.96)			
Upper 1/3	1.43 (0.83–2.47)			
Gastric linitis	11.52 (1.49–88.75)			
Type of surgery		0.02		
Subtotal gastrectomy	1			
Total gastrectomy	1.84 (1.16–2,92)			
Others	1.83 (0.65–5.10)			
Lymphadenectomy		0.01		
D1	1			
D2	0.52 (0.32–0.86)			
Lauren type		0.18		
Intestinal	1			
Diffuse	1.44 (0.86–2.40)			
Mixed	1.57 (0.91–2.71)			
pT		< 0.001		
pT1	1.00			
pT2	1.20 (0.47–3.05)			
pT3	2.58 (1.10–6.02)			
pT4	6.62 (2.24–19.5)			
pN		** < 0.001**		** < 0.001**
pN0	1.00		1.00	
pN1	1.29 (0.63–2.66)		1.66 (0.85–3.27)	
pN2	1.46 (0.72–2.95)		1.48 (0.75–2.92)	
pN3a	2.97 (1.49–5.93)		2.93 (1.48–5.78)	
pN3b	5.65 (2.89–11.03)		6.42 (3.25–12.70)	
Postoperative complications		** < 0.001**		** < 0.001**
No	1.00		1.00	
Yes	2.81 (1.85–4.27)		3.04 (1.97–4.70)	
C-D		< 0.001		
0	1.00			
I–II	2.62 (1.54–4.47)			
III–IV	3.36 (1.98–5.70)			
V	49.48 (20.98–117.12)			
Infectious complications		0.56		
No	1			
Yes	1.44 (0.41–4.98)			
NLR		**0.01**		**0.04**
< 2.4	1		1	
≥ 2.4	1.77 (1.14–2.75)		1.55 (1.02–2.37)	

*HR*, hazard ratio; *C-D*, Clavien–Dindo classification; *NLR*, neutrophil-to-lymphocyte ratio

Multivariant analysis showed that lymph node metastases (pN) (*p* < 0.001), presence of postoperative complications (*p* < 0.001) and NRL ≥ 2.4 (*p* = 0.04) were independently associated with poor survival (Table 3).

## Discussion

This study shows that the preoperative systemic inflammatory response of the host correlates with overall survival independently in patients with curative gastric cancer resection. Also, postoperative complications and their severity were associated with the worst prognosis, but the inflammatory response of postoperative complications (which differs from the inflammation produced by the oncological process) did not affect preoperative NLR.

The prognostic value of preoperative NLR has previously been reported for gastric cancer and other types of tumours [2, 4, 21–27]. It is well known that peripheral blood neutrophils, lymphocytes, monocytes and platelets, as well as their combination by ratios, behave as inflammatory markers in patients with cancer and play a relevant role in tumour-related immunity. However, the relationship between blood levels and the local inflammatory tumour microenvironment has not been established to date.

Neutrophils could promote growth and metastasis of tumours through secreting cytokines, chemokines and vascular endothelial growth factor and promote adhesion between circulating tumoral cells and distant organs, increasing the chance of distant metastases [28]. Moreover, neutrophils could also inhibit the antitumour immune function of the Natural Killer and cytotoxic T cells. Lymphocytes play a relevant role in tumour-related immunity. Several subtypes of tumour infiltrating lymphocytes such as CD8 + T cells and memory T cells are associated with better outcomes, but some subsets of T cells, regulatory T cells and Th17 cells are related to tumour progression and unfavourable prognosis. However, a high level of absolute lymphocytes count in blood is associated with an antitumour function, inhibition of tumour progression and favourable prognosis [29].

This study showed that pT, pN, type of surgery, type of lymphadenectomy, NLR and severity of postoperative complications (C-D) were predictors of long-term survival. However, multivariate analysis showed that only pN, postoperative complications and NLR remained independent prognostic factors. These findings underline that systemic inflammatory status has an important influence on the prognosis of patients with gastric cancer, independently from tumour stage and the presence of POC, suggesting that NLR can behave as a reliable marker of the host inflammatory status against the tumour.

However, it has been described [30, 31] that an altered preoperative inflammatory state of the patient can favour POC and these POC (especially the infectious and severe ones) are related to a higher risk of tumour recurrence. It could be argued that only morbidity itself has a real influence on long-term prognosis rather than the preoperative systemic inflammatory state [10, 11, 30].

Our results suggest that the mechanisms through which preoperative systemic inflammatory response (NLR ≥ 2.4) influences overall survival were not mediated through the development of surgical complications. Both parameters were independent prognostic factors. This is an important concept because POC are not uncommon after gastric cancer surgery and affect almost 40% of patients in this study, the most common being anastomotic leaks (8.8%) and intraabdominal abscess (8.8%). These results are in concordance with recent studies [32].

Our results also show the importance of the preoperative value of NLR in the prognosis and its influence on the overall survival of different anatomopathological stages (TNM classification). NLR significantly influenced stages III and IV so that patients with the same TNM stage had different overall survival according to NLR value. This phenomenon could be explained because most of the patients of our cohort had an advanced TNM stage. This is an important observation because it allows us to differentiate those patients with the same TNM stage and different long-term survival [33, 34].

It is known that surgery and non-infectious complications (like obstruction, perforation and haemorrhage) are associated with the generation of systemic inflammatory response, resulting in the suppression of cell-mediated immunity [35]. The development of postoperative infectious complications results in an up-regulation of innate immune response and the suppression of adaptive immunity, favouring an increased risk of recurrence [30].

Previous hypotheses relating to surgical complications and a reduction in survival were based on the paradigm that infective complications initiate an inflammatory cascade, activate pro-inflammatory cytokines and vascular growth factors which promote tumour growth and dissemination [36]. Although the mechanisms are not well known, the systemic inflammatory response induced by the tumour may differ from that induced by surgical trauma or infection. An elevated preoperative NLR has been associated with a greater density of CD4 + lymphocytes around the tumour, without other specific immune cells, like CD3 + or CD8 + lymphocytes, suggesting a relationship between peripheral inflammatory response and immune activation in the local tumour microenvironment [37]. In our series, we found more infectious POC in the NLR ≥ 2.4, in line with previous studies [31, 38], but this phenomenon did not correlate with differences in overall survival.

Taking all these reasons into account, we should differentiate preoperative tumour-related inflammation from the inflammatory mechanisms of POC, which will influence prognosis in different ways.

Another important issue in most studies is the lack of consensus on the cut-off value for NLR. One of the controversies of this type of study is the lack of standardisation to determine the cut-off value for NLR, ranging in the literature from 2 to 5 [33, 39–41]. We used an NLR cut-off value of 2.4, based on the median value, with similar results to other studies [9, 42]. However, different methods can be used to calculate NLR, such as ROC curve, median value or the use of computer X-Tile software [29]. Because of this variability in the cut-off values, it is difficult to use preoperative NLR as a clinical standardised prognostic value. Besides, a combination of different preoperative systemic inflammatory markers (NLR, LMR, total number of monocytes and lymphocytes) may provide a better predictive value than each one alone, but this has not been confirmed to date [43].

The limitations of this study include those related to its retrospective design, the limited number of patients, the impossibility of having a standard cut-off value for NLR and that the prognostic value of peripheral blood cells after surgery was not evaluated. More studies are needed to establish a clear cut-off value of preoperative inflammatory markers and help to find its utility in clinical practice.

In conclusion, the preoperative systemic inflammatory response in patients with gastric cancer, measured by neutrophil-to-lymphocyte ratio, behaves as an independent prognostic factor, even in those patients with postoperative complications. More prospective trials are necessary to validate these data.

## Data Availability

Not applicable.
